# Transient mTOR Inhibition Facilitates Continuous Growth of Liver Tumors by Modulating the Maintenance of CD133+ Cell Populations

**DOI:** 10.1371/journal.pone.0028405

**Published:** 2011-12-01

**Authors:** Zhaojuan Yang, Li Zhang, Aihui Ma, Lanlan Liu, Jinjun Li, Jianren Gu, Yongzhong Liu

**Affiliations:** 1 State Key Laboratory of Oncogenes and Related Genes, Shanghai Jiao Tong University School of Medicine, Renji Hospital, Shanghai Cancer Institute, Shanghai, China; 2 Institute of Reproductive Immunology, Jinan University, Guangzhou, China; Roswell Park Cancer Institute, United States of America

## Abstract

The mammalian target of the rapamycin (mTOR) pathway, which drives cell proliferation, is frequently hyperactivated in a variety of malignancies. Therefore, the inhibition of the mTOR pathway has been considered as an appropriate approach for cancer therapy. In this study, we examined the roles of mTOR in the maintenance and differentiation of cancer stem-like cells (CSCs), the conversion of conventional cancer cells to CSCs and continuous tumor growth *in vivo*. In H-Ras-transformed mouse liver tumor cells, we found that pharmacological inhibition of mTOR with rapamycin greatly increased not only the CD133+ populations both *in vitro* and *in vivo* but also the expression of stem cell-like genes. Enhancing mTOR activity by over-expressing Rheb significantly decreased CD133 expression, whereas knockdown of the mTOR yielded an opposite effect. In addition, mTOR inhibition severely blocked the differentiation of CD133+ to CD133- liver tumor cells. Strikingly, single-cell culture experiments revealed that CD133- liver tumor cells were capable of converting to CD133+ cells and the inhibition of mTOR signaling substantially promoted this conversion. In serial implantation of tumor xenografts in nude BALB/c mice, the residual tumor cells that were exposed to rapamycin *in vivo* displayed higher CD133 expression and had increased secondary tumorigenicity compared with the control group. Moreover, rapamycin treatment also enhanced the level of stem cell-associated genes and CD133 expression in certain human liver tumor cell lines, such as Huh7, PLC/PRC/7 and Hep3B. The mTOR pathway is significantly involved in the generation and the differentiation of tumorigenic liver CSCs. These results may be valuable for the design of more rational strategies to control clinical malignant HCC using mTOR inhibitors.

## Introduction

Cancer stem-like cells (CSCs) are the most malignant subpopulations in tumors because of their resistance to drug therapy and association with poor outcomes. As in adult tissue-specific stem cells, which have extensive self-renewal abilities that ensure the continuous turnover of normal tissues, CSCs are required for continuous tumor growth. Great progress has been made in the identification and characterization of CSCs in several types of solid tumors, such as those of the brain, breast, colon and liver [1]–[7]. In addition, a few key signaling pathways, including Wnt/β-catenin, AKT and TGF-β [8]–[13], have been implicated in the maintenance of CSCs. However, the molecular events that preserve the pool size and stem cell properties of CSCs by maintaining the balance of proliferation, differentiation and self-renewal are still poorly understood. Elucidating the mechanistic differences between CSCs and conventional tumor cells may be valuable for developing strategies for cancer therapy.

The mammalian target of rapamycin (mTOR) is an essential serine/threonine kinase that regulates cell growth by controlling protein synthesis, autophagy, endocytosis, and metabolism in response to growth factors, nutrients, energy, and stress [14]. Increasing evidence indicates that the signaling pathways that activate mTOR are frequently improperly regulated in most human cancers [15]–[23]. Rapamycin, an inhibitor of mTOR, can block tumor growth and inhibit tumor cell motility [24]–[26]. These findings have promoted the clinical use of mTOR inhibitors for cancer therapy. However, the use of mTOR inhibitors alone has had limited clinical success [27], [28]. The role of mTOR signaling in the maintenance of CSCs has been addressed recently, but the conclusions of these reports are controversial. In embryonic and adult stem cells, mTOR hyperactivation resulted in the differentiation and exhaustion of stem cells [29]–[32]. In the tumor development, mTOR signaling has been shown to enhance the survival of dormant tumor cells [33] and maintain the self-renewal and tumorigenicity of glioblastoma stem-like cells [34] and breast cancer stem-like cells [35]. In sharp contrast, mTOR inhibition by rapamycin has been shown to significantly increase CD133 expression in gastrointestinal cancer cells via down-regulation of HIF-1α [36]. However, there is a lack of *in vivo* data regarding the changes in CSC maintenance and tumorigenicity in tumor cells after transient exposure to rapamycin.

In this study, we examined the role of mTOR in the regulation of CD133+ CSCs differentiation, the conversion of CD133- conventional cancer cells to CD133+ CSCs, and the repopulation *in vivo* of mTOR-manipulated tumor cells. We found that mTOR inhibition increased the CD133+ subpopulation in liver tumor cells and potentiated the continuous growth of tumor cells *in vivo* via preventing differentiation, biased insensitivity of CD133+ subpopulation to rapamycin-induced apoptosis, and increasing the retrodifferentiation of CD133- to CD133+ cells.

## Results

### mTOR inhibition increases CD133+ subpopulations and retain stemness properties in liver tumor cells

**Figure 1:**
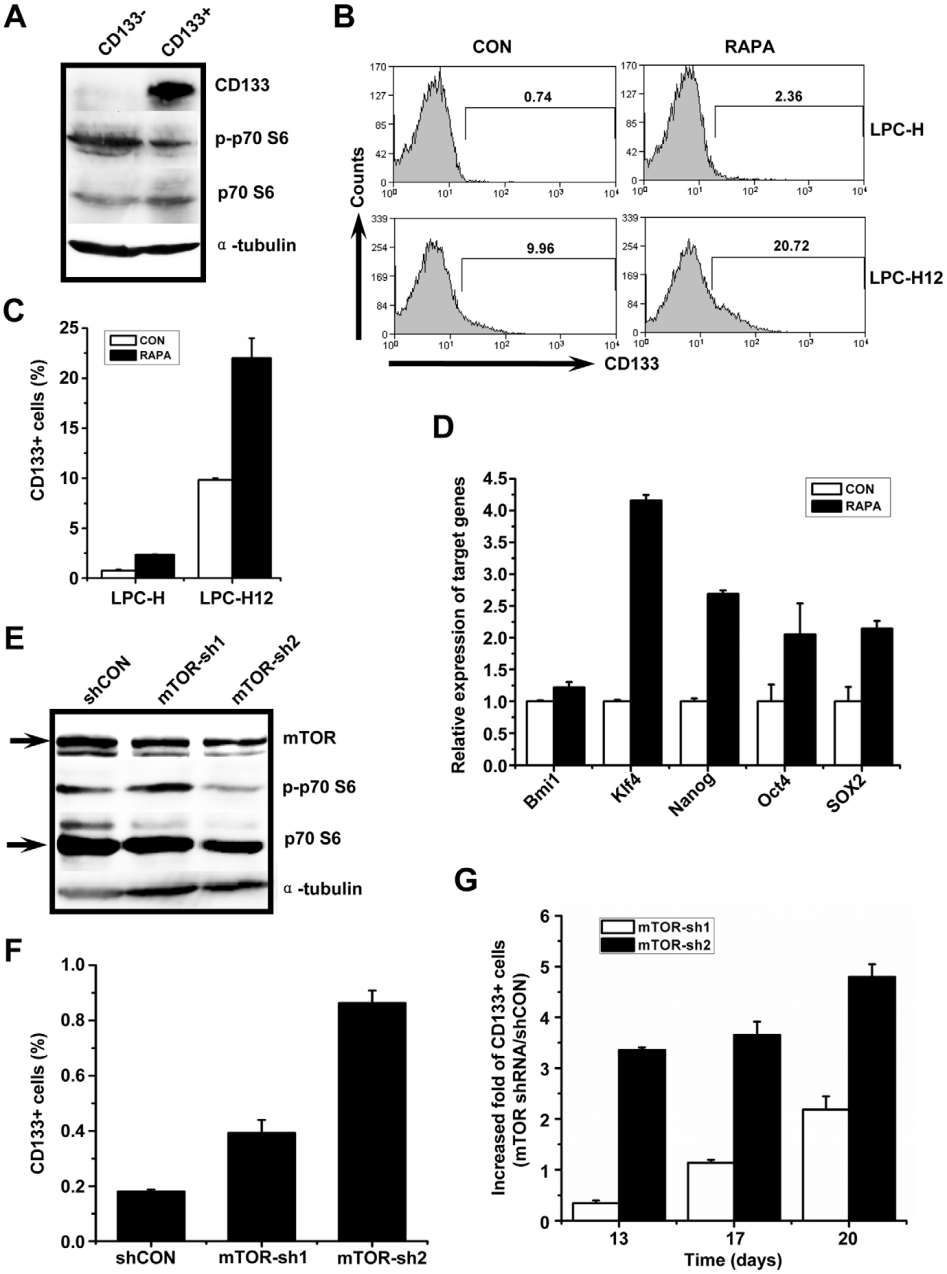
mTOR inhibition enriches CD133+ populations in oncogenic H-Ras-transformed mouse liver tumor cells. **A** mTOR signaling was highly activated in CD133- subpopulations when compared with CD133+ compartments. Immunoblotting was performed for CD133, phosphorylated p70 S6 (p-p70 S6), p70 S6, and α-tubulin as a loading control. **B, C** Enrichment of CD133+ cells in LPC-H and LPC-H12 cells after rapamycin (RAPA) treatment (10 nM) for 3 days. Representative FACS profiles (B); summary bar graphs illustrate percentage of CD133+ cells as means±S.E.M. of the results from three experiments (C). **D** Stem cell-associated gene expression measured via qPCR in LPC-H12 cells treated with or without rapamycin (10 nM for 24 h). Real-time PCR experiments were conducted in triplicate and with GAPDH for expression normalization. **E** Immunoblotting for mTOR, p-p70 S6, and p70 S6 confirmed mTOR knockdown by shRNAs. **F, G** Sustained increases of CD133 expression were observed in LPC-H cells stably expressing shRNAs targeting mTOR. The means±S.E.M. of the percentages of CD133+ cells in LPC-H cells infected with retrovirus expressing mTOR-shRNA (n = 3) are shown in F. The magnitude of the change in percentage of CD133+ cells in LPC-H on days 13, 17 and 20 after infection with mTOR-sh1 and mTOR-sh2 containing retrovirus (means±S.E.M., n = 3) (G).

To elucidate whether mTOR is involved in the maintenance of CSCs, H-Ras-transformed mouse liver tumor cells were employed in the study. We found that the percentage of CD133+ cells, which have been referred as a subpopulation containing CSCs [5], [37], [38], was approximately 0.2 to 1% in H-Ras-transformed mouse liver tumor cells (LPC-H) and 10 to 20% in the derived clone LPC-H12, both of which expressed markers of live progenitor cells, and the latter displayed much high potential of tumorigenicity (Figure S1). To examine whether there was a significant difference in mTOR activation between CD133- and CD133+ LPC-H12 cells, we purified these two population by FASC sorting and found that the CD133+ subsets exhibited lower activation of mTOR signaling compared with CD133- cells, as indicated by the levels of phosphorylated p70 S6 kinase (p-p70 S6) (Figure 1A). We next examined whether mTOR inhibition could lead to a change in the proportion of CD133+ LPCs. LPC-H and LPC-H12 cells were treated with the mTOR inhibitor rapamycin and measured CD133 expression by FACS. Intriguingly, the CD133+ fraction of the cells treated with rapamycin was significantly greater than that of the cells without treatment (Figure 1B and 1C). The influence of rapamycin on the maintenance of CD133+ subpopulations was dose- and time-dependent (Figure S2A and S2B). Expression of p-p70 S6 was undetectable in cells after rapamycin treatment, demonstrating the efficiency of rapamycin in eliminating mTOR activity (Figure S2C). Notably, we found that the expression of several stem cell-associated genes, such as Nanog, Klf4 and SOX2, were increased significantly after treatment with rapamycin (Figure 1D). To further identify the role of mTOR in the CD133+ subpopulation, we knocked down mTOR expression in LPC-H cells using the retrovirus expressing short hairpin RNA (shRNA) (Figure 1E). Downregulation of mTOR expression via mTOR shRNA-2 (mTOR-sh2) dramatically increased the CD133+ fraction in cells in a time-dependent manner (Figure 1F and 1G). Similar results were obtained in LPC-H12 cells (Figure S2D).

**Figure 2:**
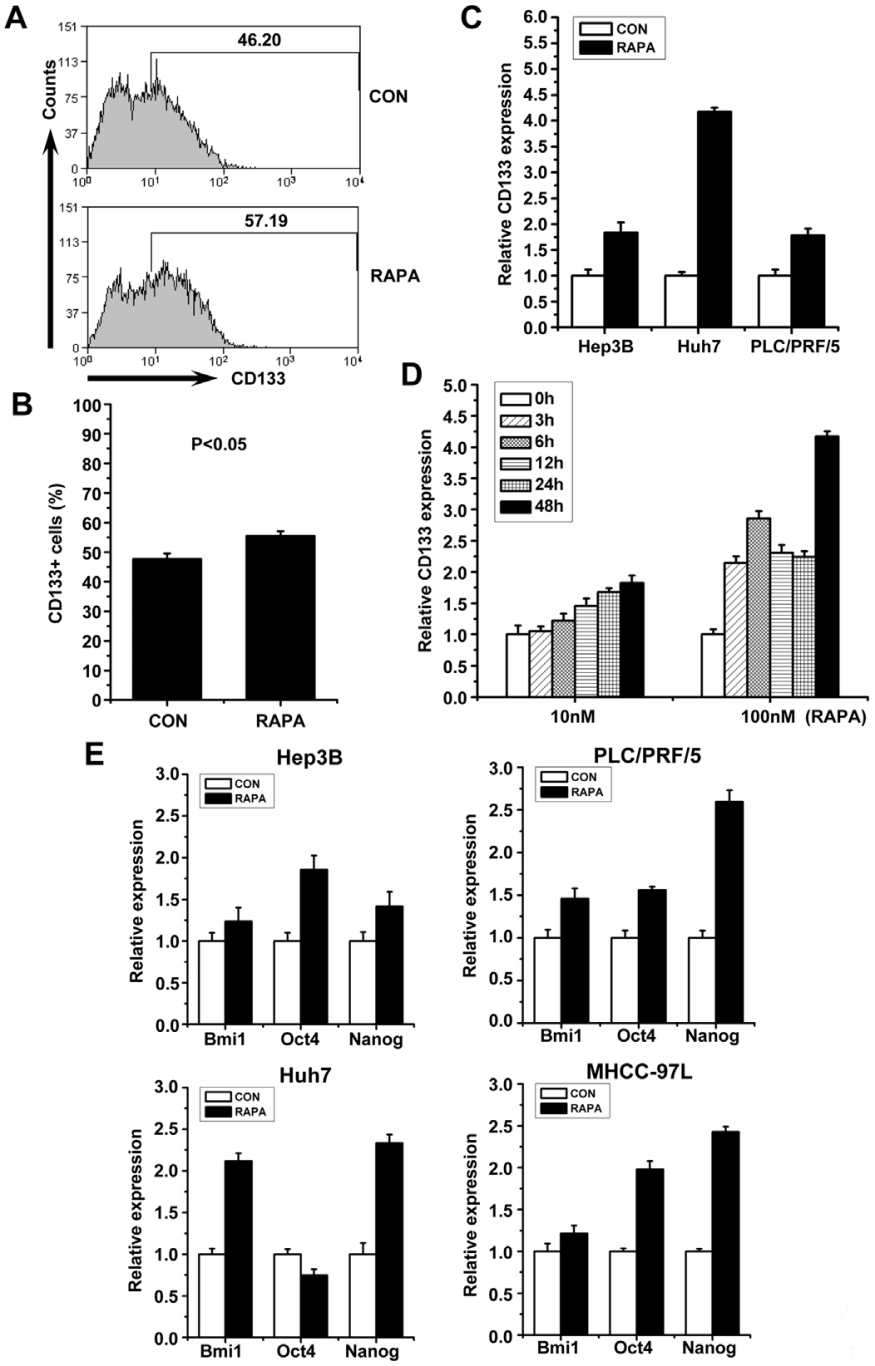
Repression of mTOR enhanced stem cell-like features of human liver tumor cells. **A, B** Enhanced CD133+ subpopulations in Huh7 cells with treatment of rapamycin (100 nM) for 5 days. Representative FACS profiles (A); Percentages of CD133+ cells are presented as means±S.E.M. (n = 3) (B). **C, D** qPCR analysis shows enrichment of CD133 expression in human liver tumor cell lines after exposed to rapamycin (100 nM). Relative expression of CD133 in Hep3B, Huh7, and PLC/PRF/5 with or without treatment of rapamycin for 48 h is shown in C. Sustained increase of CD133 expression in Huh7 after treatment of rapamycin with 10 nM or 100 nM is illustrated in D. The sustained GAPDH was used as an internal control. **E** qPCR ananlysis was performed for stem cell-associated genes in multiple human liver tumor cell lines. Cells without treatment of rapamycin (100 nM) were defined as 1, and the relative expression of each gene in cells with treatment of rapamycin for 48 h was expressed as the fold difference over this baseline. GAPDH was used as an internal control.

In some human liver tumor cell lines, such as Hep3B, Huh7 and PLC/PRF/5, the enhanced lever of CD133 expression after treatment with rapamycin was also detected (Figure 2A–2D). Similarly, the expression of stem cell-associated genes, including Bmi1, Oct4 amd Nanog, were significantly increased in the human liver tumor cell lines treated with rapamycin, even in MHCC-97L cells that are no CD133 expression (Figure 2E). Collectively, these data indicate that inhibition of mTOR could enrich CSCs in both mouse and human liver tumor cells.

To investigate whether the disparities exist in the sensitivity to rapamycin between CD133- and CD133+ cells, we directly examined the effect of rapamycin on cell growth of the two subsets among LPC-H12 cells. We found that rapamycin-mediated inhibition in CD133- cells was greater than that in CD133+ cells (Figure S3), indicating that a greater impairment of the CD133- subset upon rapamycin treatment might partially contribute to the enrichment of CD133+ cells.

### mTOR activation by Rheb overexpression decreases the CD133+ subpopulations

**Figure 3:**
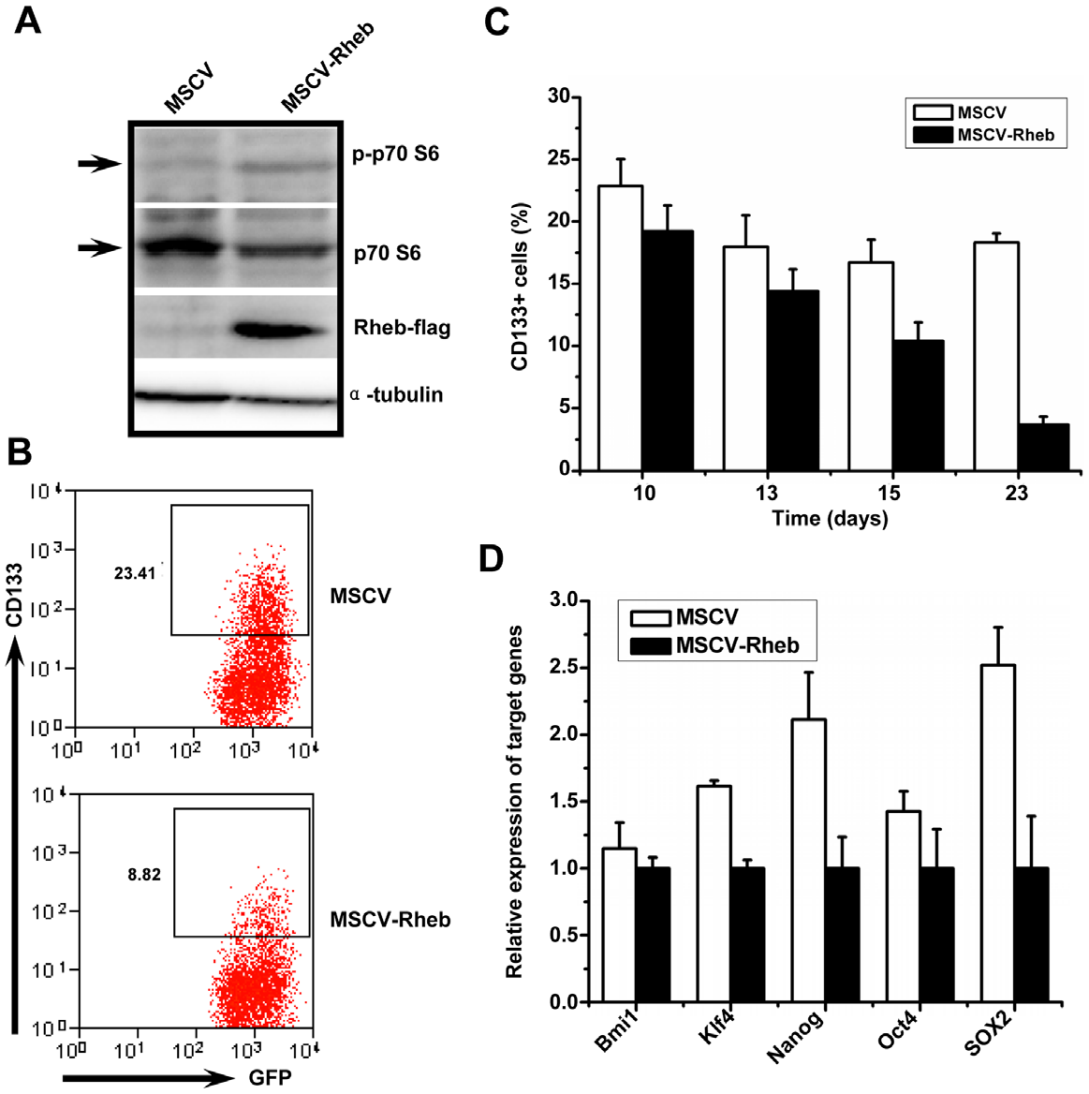
Activation of mTOR signaling by overexpression of Rheb inhibits the CD133 expression and stem cell-like potential. **A** mTOR signaling was activated by overexpression of Rheb. Immunoblotting was performed for expression of p-p70 S6, p70 S6, and Rheb; α-tubulin was used as a loading control. **B, C** Reduction in the pool size of CD133+ compartments following Rheb overexpression in LPC-H12 cells. LPC-H12 cells were infected with MSCV-Rheb or control MSCV retrovirus. At different time points, the cells were stained with mouse CD133-PE antibody to identify CD133 expression. Representative FACS profiles of CD133 expression in LPC-H12 cells on day 12 after retroviral infection (B). Summary bar graphs of the proportion of CD133+ cells in LPC-H12 after infection with MSCV-Rheb or control retrovirus (means±S.E.M., n = 3) (C). **D** The magnitude of change in stem cell-associated gene expression measured via qPCR in LPC-H12 cells with over-expression of Rheb. Real-time PCR experiments were conducted in triplicate and with GAPDH for expression normalization.

To test whether activation of mTOR signaling could decrease the CD133+ subpopulations, Rheb, an activator of mTOR signaling [39], was overexpressed in LPC-H and LPC-H12 cells by retroviral infection. We found that Rheb expression significantly enhanced mTOR activity, as determined by increased phosphorylation of p70 S6 (Figure 3A). As expected, mTOR activation resulted in significant exhaustion of the CD133+ subsets (Figure 3B). A time-course analysis of CD133 expression by FACS showed that Rheb overexpression gradually decreased the percentage of CD133+ cells in LPC-H12 cells (from 19.22±2.09% to 3.94±0.86%), whereas cells infected with empty retrovirus showed only a slight decrease in CD133 expression (from 22.85±2.17% to 16.99±1.28%) (Figure 3C). Furthermore, the expression of all detected stem cell-associated genes were significantly reduced in Rheb-overexpressed cells (Figure 3D). These results suggested that mTOR activation might enhance the differentiation of CD133+ to CD133- liver tumor cells.

### Single-cell culture experiments define a critical role for mTOR in the conversion of CD133- to CD133+ liver tumor cells

**Figure 4:**
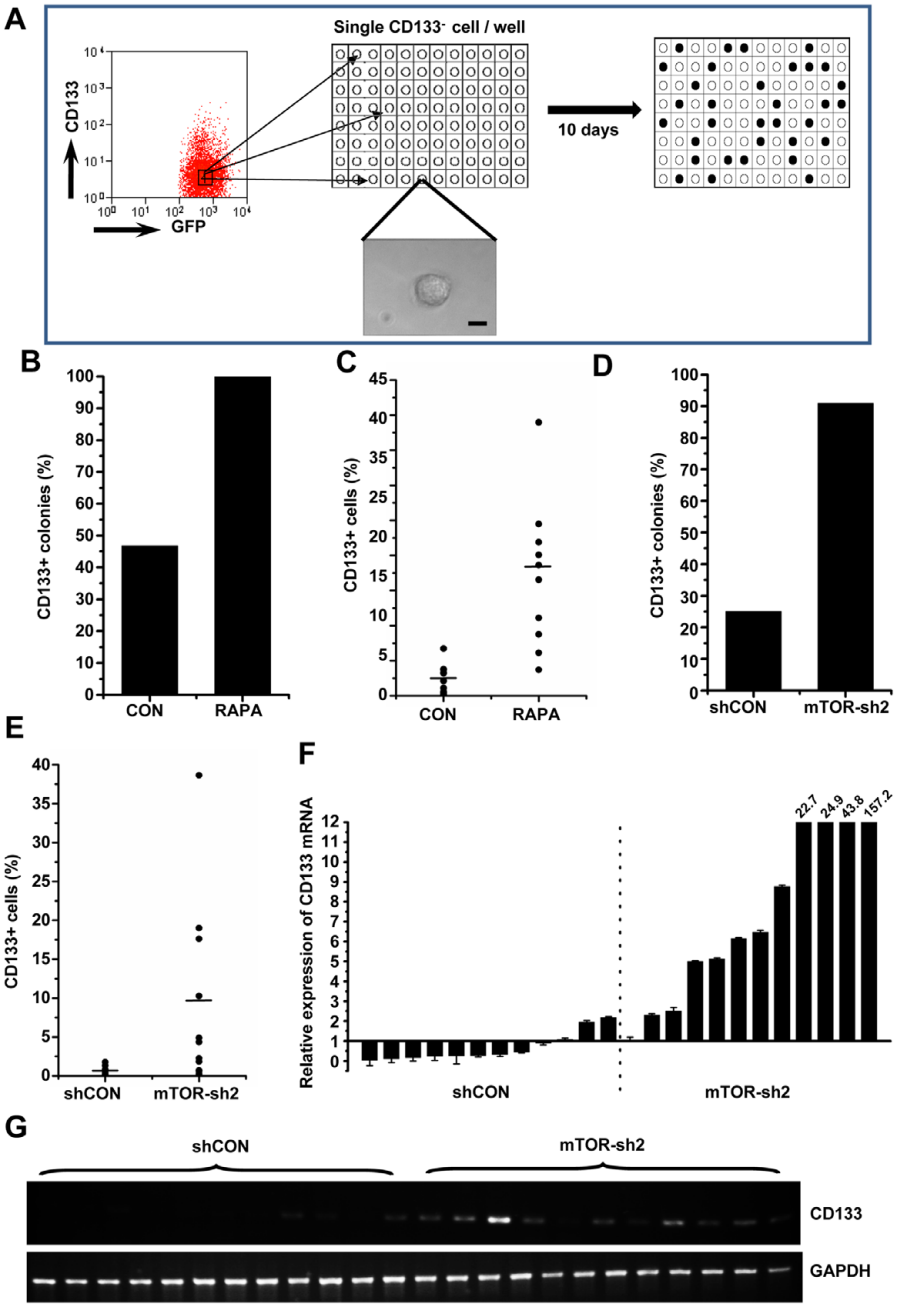
mTOR inhibition facilitates the conversion of CD133- to CD133+ cells. **A**–**C** De novo generation of CD133+ cells after long-term single-cell culture of CD133- LPC-H12 cells. Single CD133- LPC-H12 cells were deposited by FACS into each well of 96-well plates with or without rapamycin (10 nM). After expansion, the single-cell derived LPC-H12 cells were analyzed by FACS. Schematic diagram shows FACS isolation and robotic plating of single CD133- cells into 96-well plates (A). Phase image of a single cell in one well after sorting (bottom). Bar = 10 µm. The percentages of the colonies containing CD133+ cells are shown in B (n = 10 per group). The proportions of CD133+ cells in each colony are shown in C. **D-G** Knockdown of mTOR expression enhanced the rate of conversion of CD133- to CD133+ cells. The percentages of CD133+ colonies derived from single CD133- LPC-H12 cells infected with retrovirus containing mTOR-sh2 and control, respectively, are shown in D (n = 12 per group). The proportions of CD133+ cells in each colony are shown in E. The expression of CD133 mRNA in each clone examined by Real-time PCR experiments in triplicate, and all samples were normalized to GAPDH expression (F). The result of RT-PCR is shown in G.

Previous studies have shown that conventional cancer cells can be induced by certain events to express stem cell markers and acquire properties of CSCs [40], [41]. To address whether CD133- cells can be converted to CD133+ tumor cells without adding exogenous TGF-β, and if so, whether mTOR signaling is involved in the process, we employed a single-cell long-term culture system (Figure 4A). CD133- cells of LPC-H12 were FACS-sorted, and each single cell was robotically plated into individual wells of 96-well plates. After 10 days in 96-well plates, single-cell derived clones were individually trypsinized and seeded into 24-well plates for expansion. After 11 days, the cells were harvested for FACS analysis of CD133 expression. Of significant interest, 46.7% of colonies derived from single CD133- LPC-H12 cells expressed CD133, and the percentage of CD133+ cells in the positive colonies was approximately 1 to 7%. Notably, we found that rapamycin treatment significantly increased both the formation of CD133+ cell colonies and the percentage of CD133+ cells in positive colonies (Figure 4B and 4C). Accordingly, using the same protocol, downregulation of mTOR expression by shRNA in the LPC-H12 cells dramatically increased the number of colonies that contained CD133+ cells and the proportion of CD133+ cells in the positive colonies (Figure 4D and 4E). Similar results were obtained using LPC-H cells (data not shown). In agreement with the FACS results, the level of *CD133* mRNA in the single-cell-derived colonies expressing mTOR-sh2 was significantly higher than that in the colonies containing control shRNA (Figure 4F and 4G).

### Repression of mTOR inhibits differentiation of CD133+ liver tumor cells

**Figure 5:**
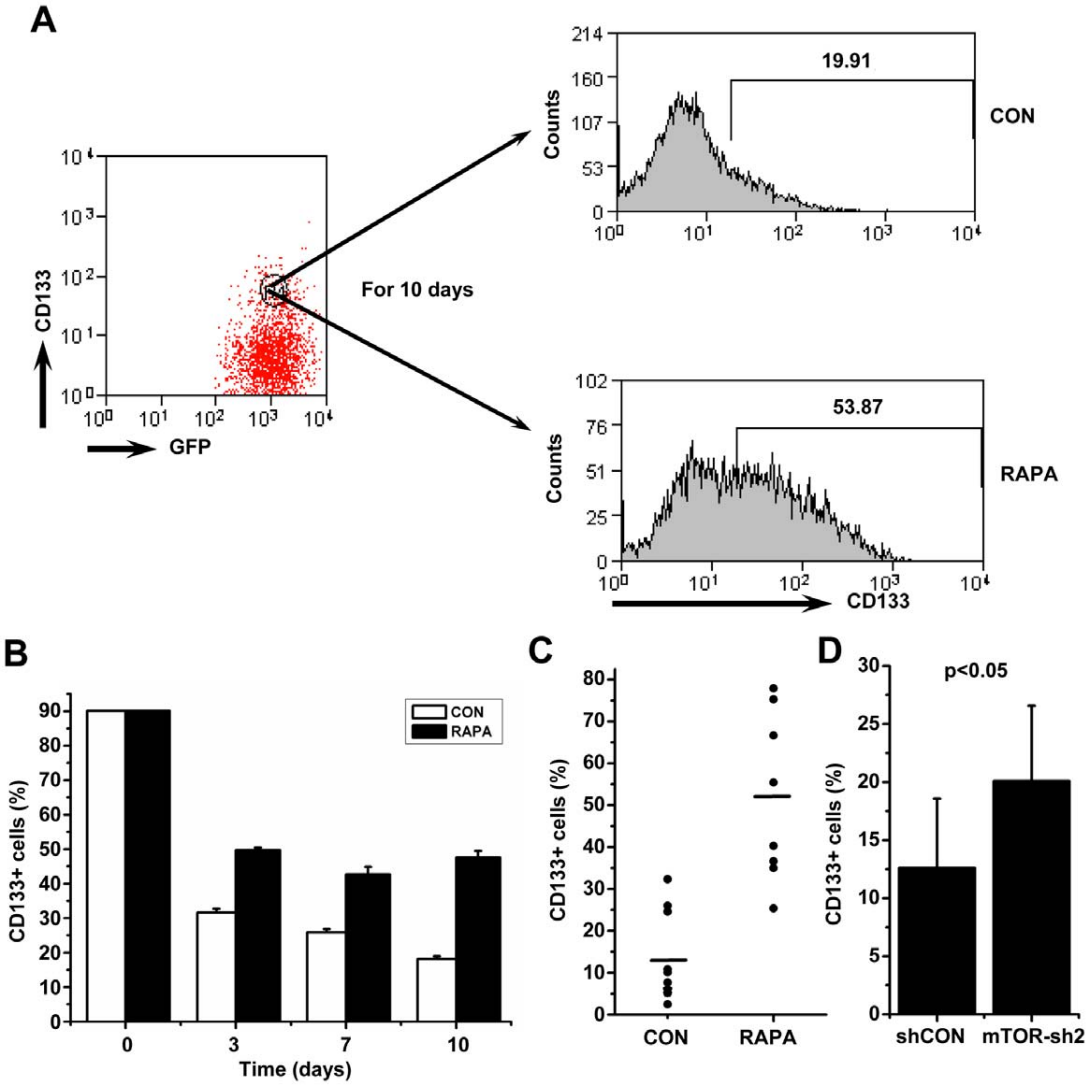
The consequences of mTOR repression on the maintenance of CD133 expression. **A, B** Inhibition of mTOR signaling by rapamycin severely blocked the rapid decrease of the CD133+ subsets over time in sorted CD133+ LPC-H12 cells. Representative FACS profiles of LPC-H12 cells are shown in A. Changes of CD133 expression in LPC-H12 cells at days 3, 7, and 10 after cell sorting are shown in B. Data shown are the means±S.E.M. of the results of three experiments. **C** Single-cell culture for analysis of the effect of rapamycin on the maintenance of the CD133+ subpopulations. The single CD133+ cell-derived colonies cultured in the media with rapamycin had more CD133+ cells than those in rapamycin-free media. Single CD133+ LPC-H12 cells were sorted into 96-well plates and cultured with or without rapamycin (10 nM). Each symbol represents an individual single-cell-derived colony; small horizontal lines indicate the means (n = 8). **D** The percentage of CD133+ cells in each colony derived from single CD133+ LPC-H12 stably expressing mTOR-sh2 was higher than that from single CD133+ LPC-H12 infected with control retrovirus. Data are shown as the means±SD (n = 8).

On the basis of recent findings that mTOR activity mediates the exhaustion and differentiation of stem cells [29]–[31], we hypothesized that the increase in the proportion of CD133+ cells in the presence of rapamycin might be partially due to blockage of differentiation of CD133+ cells. To test this hypothesis, we isolated CD133+ cells from LPC-H12 by FACS sorting, and further treated cells with rapamycin for 10 days. Strikingly, we found that CD133+ expression declined substantially in the control group, whereas the proportion of CD133+ cells in the rapamycin-treated group was maintained (Figure 5A and 5B). Moreover, we sorted CD133+ cells and seeded them for single-cell culture. At day 10 of culture, 32.8% (63 of 192 wells) and 54.6% (105 of 192 wells) single-cell-derived colonies were formed in the culture medium with or without rapamycin, respectively. These colonies were then transferred into 24-well plates. After one week of culture, we found high levels of CD133+ cells in the colonies that had been treated with rapamycin (Figure 5C). In agreement with these results, high CD133 expression was associated with colonies derived from single CD133+ cells with expression of shRNAs against mTOR (Figure 5D). Collectively, these results illustrate that inhibition of mTOR can maintain CD133+ subpopulations by suppressing the differentiation of CD133+ cells.

### mTOR inhibition maintains CD133+ subpopulations *in vivo* and enhances re-propagation of Ras-dependent liver tumors

**Figure 6:**
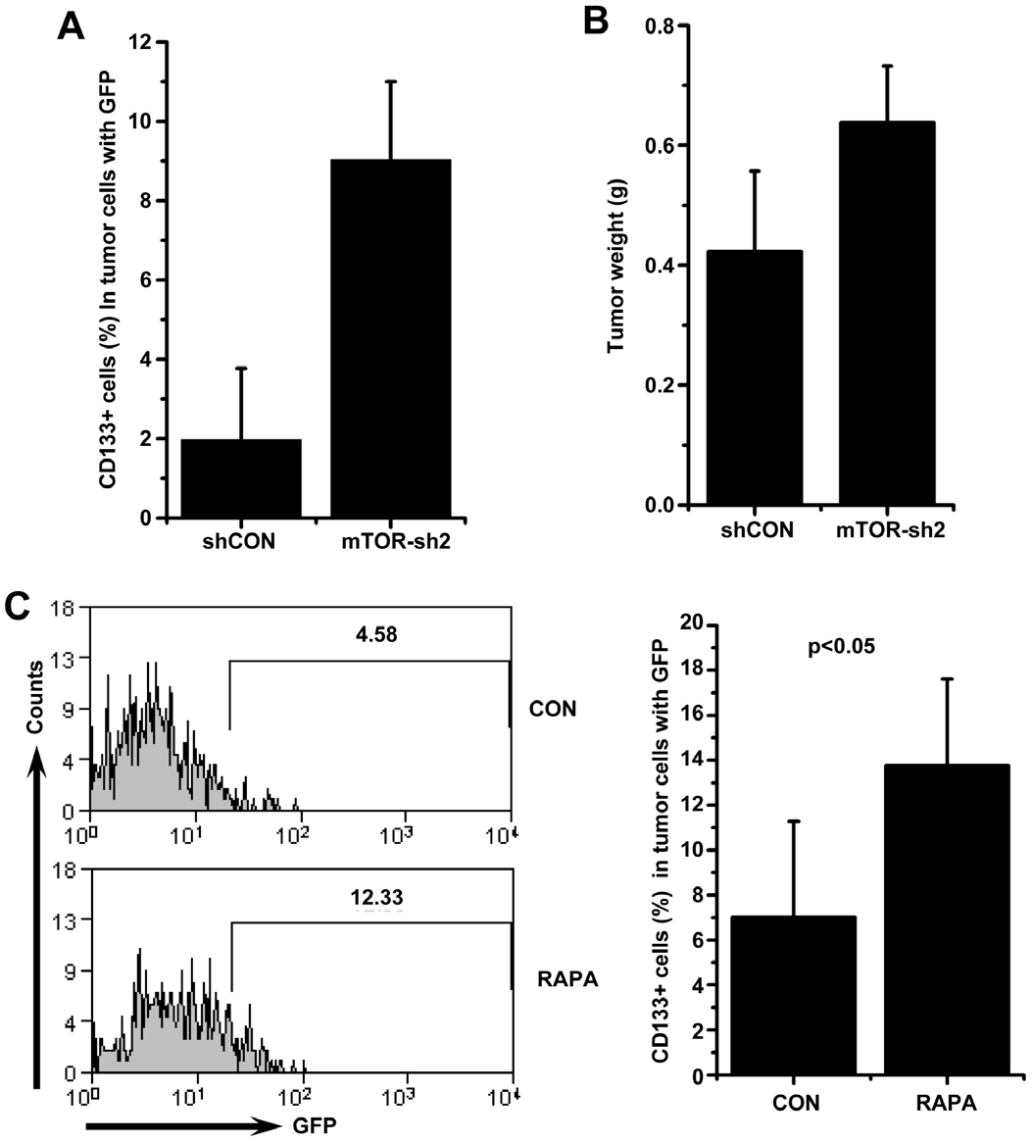
Inhibition of mTOR increases the CD133+ cells *in vivo*. **A** The percentage of CD133+ cells in GFP+ cells harvested from xenograft tumors with mTOR-sh2 expression was higher than that from control ones. LPC-H cells with GFP infected with control or mTOR-sh2 retroviral vector were subcutaneously implanted into nude BALB/c mice (n = 3 per group). The animals were euthanized on day 24. The percentage of CD133+ cells in GFP+ tumor cells with expression of mTOR shRNA and control shRNA are shown as the means±SD (n = 3). **B** There was no significant effect of knockdown mTOR on tumor growth of LPC-H cells in vivo. Differences in tumor size in mm^3^ between control and mTOR-knockdown groups are reported as means±SD. **C** The xenografts of LPC-H cells contained a high proportion of CD133+ cells in the mice after rapamycin treatment for 10 days. FACS analysis profile (left). Percentages of CD133+ cells in LPC-H cells in vivo are presented as means±SD (n = 5) (right).

To assess the maintenance of CD133+ subpopulations in tumor cells *in vivo* by inhibition of mTOR, we injected LPC-H cells expressing mTOR-sh2 or control shRNA into nude mice. After two weeks, both types of cells resulted in tumor formation. However, using FACS analysis to detect GFP+ tumor cells in the xenotransplants on day 24, a high percentage of CD133+ cells were preserved in tumors derived from LPC-H cells expressing shRNA against mTOR (Figure 6A). No significant difference was found in tumor size between the two types of cells (Figure 6B). Increased proportion of CD133+ tumor cells was also found in the tumor tissues after treatment of the tumor-bearing mice with rapamycin for 10 days (Figure 6C).

**Figure 7:**
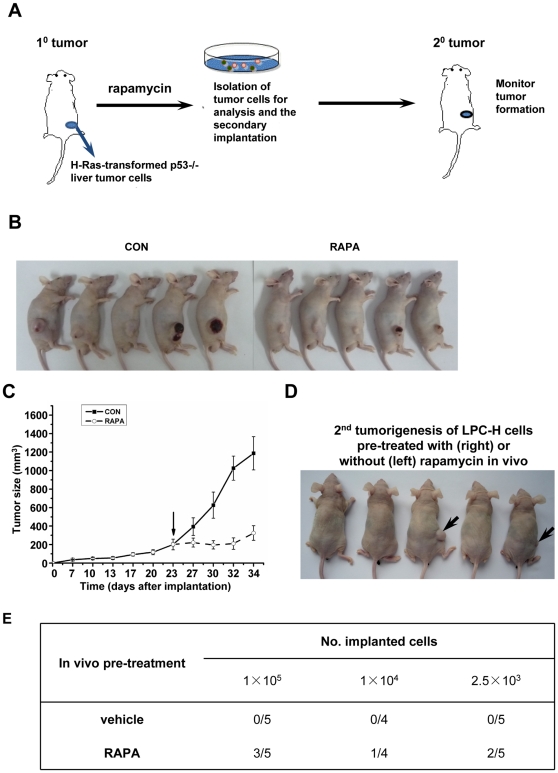
Administration of rapamycin facilitates continuous tumorigenesis. **A** Serial xenograft strategy. LPC-H cells were subcutaneously injected into nude BALB/c mice (n = 5 per group). After 3 weeks of tumor growth, the animals were treated with rapamycin (1.5 mg/kg/day) or diluents for 10 days, and then euthanized. Tumor cells were dissociated, and GFP+ tumor cells were then implanted into secondary recipients. **B, C** Subcutaneous tumors grew more slowly in nude BALB/c mice after treatment with rapamycin than did tumors without rapamycin treatment. Original magnification (B) and growth curve of xenograft tumors in nude mice (C). **D** Continuous tumorigenesis of LPC-H cells with exposure to rapamycin in vivo was more efficient than that without rapamycin treatment. The tumor cells from nude BALB/c mice with or without treatment with rapamycin (1.5 mg/kg/day) for 10 days were secondarily implanted into the right or left flanks of the mice, respectively. Arrows indicate tumor formation (2.5×10^3^ GFP+ tumor cells implanted). **E** The secondary tumor-initiating ability of LPC-H cells treated with or without rapamycin in the nude BALB/c xenograft transplant model. Tumor initiation was monitored for 4 wk after implantation.

The increase in CD133+ subsets induced by mTOR inhibition led us to speculate that transient treatment with rapamycin might result in the enhanced continuous growth of Ras-dependent mouse liver tumors *in vivo*. We tested this hypothesis by taking advantage of the secondary tumor xenograft model (Figure 7A). GFP+ LPC-H cells were subcutaneously injected into nude mice. The nude mice bearing xenografts were then treated with rapamycin or control diluents beginning 3 weeks after tumor implantation. After 10 days of rapamycin treatment, tumor growth was significantly reduced (Figure 7B and 7C). The tumor tissues were enzymatically dissociated into single-cell suspensions, and CD133 expression was analyzed in the GFP+ LPC-H cells. The significant increase in CD133 expression in tumor cells from the first implantation after rapamycin treatment was similar to the effect of downregulation of mTOR *in vitro*. The tumor cells, harvested by FACS sorting of GFP+ population, were then transplanted into the secondary recipients. We found that as few as 2.5×10^3^ tumor cells with rapamycin treatment were sufficient to initiate tumor development, whereas up to 1×10^5^ tumor cells without rapamycin treatment failed to initiate subcutaneous tumors in secondary recipient mice (Figure 7D and 7E). Thus, mTOR inhibition by rapamycin could block tumor growth from the first implantation but enhance continuous growth of the secondary tumors derived from H-Ras-transformed mouse liver cells.

## Discussion

Aberrant activation of the mTOR pathway has been implicated in the growth of various human cancers [42], [43]. mTOR signaling promotes cell proliferation, and, in the context of cancer development, it is highly correlated with tumor expansion and rate of tumor growth. Remarkably, several reports have highlighted the critical importance of mTOR signaling in driving differentiation of adult progenitor cells accompanied by rapid cell expansion [29]–[32]. Recently, it has been established that the preservation of small subpopulations of CSCs within tumors is critical for tumors to assume malignant behavior and resistance to therapy. To date, whether mTOR signaling is critically involved in maintaining the balance between proliferation and differentiation is still poorly defined. Because mTOR inhibitors have been utilized for cancer therapy, and only modest or unremarkable success has been achieved in some clinical trials [27], [28], it is urgent to investigate the long-term effects of transient mTOR treatment on tumor re-emergence and malignant outcome. We demonstrated here that targeted inhibition of mTOR significantly enhanced the generation and maintenance of CD133+ subpopulations and promoted secondary tumor re-propagation of H-Ras-transformed mouse liver tumor cells *in vivo*.

CD133 is considered a universal marker of stem cells and CSCs [44]–[46]. CD133+ tumor cells exhibit properties of CSCs in human hepatocellular carcinomas and mouse liver cancer [47]–[52]. In this study, we found that H-Ras-transformed mouse liver tumor cells expressed the surface marker CD133. To our surprise, by exploiting the difference in the status of mTOR activation between CD133+ and CD133- populations, we found elevated mTOR signaling in the CD133- subset. Although the mTOR pathway has been reported to be important for the viability and maintenance of breast and glioblastoma CSCs [34], [35], inhibition of mTOR with rapamycin in H-Ras-transformed mouse liver cancer cells significantly increased the proportion of CD133+ cells and retained the stemness properties. Using RNAi-mediated knockdown of mTOR and overexpression of Rheb, an activator of the mTOR kinase [53], we confirmed a critical role for mTOR in exhaustion and differentiation of the CD133+ subsets. Interestingly, these data are consistent with the notion that elevated p70 S6 kinase activation induces differentiation of human embryonic stem cells (hESCs) [30]. Furthermore, a previous study has shown that persistent activation of mTOR in normal epithelial stem cells results in exhaustion of these stem cells [29]. Notably, mTOR pathway is also significantly important in the process of cellular senescence in multiple human and rodent cells [54]–[56]. Rapamycin and MEK inhibitor that can target mTOR/S6 pathway, decelerate cellular senescence. Moreover, mTOR inhibition attenuates cellular senescence but favors quiescence in p53- arrested cells. These important findings are principally supportive to our observation since the impaired capacity in proliferation and the great potential to regain propagation (rejuvenation) are basically the features of CSCs. We must note that the similar effect of rapamycin was also found in multiple human liver tumor cell lines, such as Huh7, PLC/PRF/5, and Hep3B, suggesting that, even it was able to inhibit growth of tumor, rapamycin might enrich liver cancer stem-like cells in a clinical setting.

Several studies have revealed that cellular stress (such as hypoxia), cytokines (such as TGF-β), or activation of the mTOR pathway are able to increase the expression of CSC surface markers and phenotypes in certain bulk tumor cells [36], [48], [57], [58], indicating that cell-extrinsic environmental factors may reprogram conventional tumor cells to cells with stem cell-like properties in the course of cancer development. In the present study, using single-cell culture experiments that can bring out conclusive results, we unexpectedly revealed that CD133- liver tumor cells were capable of converting to CD133+ cells, and blocking intracellular mTOR activity with rapamycin or RNAi against mTOR indeed promoted the conversion of CD133- to CD133+ cells. Notably, certain stem cell-associated genes are upregulated by rapamycin prior to the increasing of CD133 expression in LPCs, suggesting that these genes might function in the rapamycin-mediated enrichment of CD133+ subpopulations. Previous studies have shown that HIF-1α is a positive downstream target of the mTOR signaling pathway [59]. In human glioma cells, the activation of HIF-1α enhances CD133+ glioma-derived cancer stem cell expansion by increasing self-renewal activity and inhibiting cell differentiation [60]. Thus, the relationship between HIF-1α and CD133 expression in the process of conversion requires further investigation.

In human liver tumor cells, the induced CD133+ cells display significant potential for tumorigenesis [48]. Indeed, we also found that CD133 expression and certain stemness genes were upregulated with the treatment of rapamycin. The relevant significance of the regulation mediated by rapamycin on CSCs maintenance was illustrated by the *in vivo* study, in which we demonstrated that the tumor cells harvested from the first implantation with treatment of rapamycin were significantly higher in secondary tumorigenicity than those from the control, as manifested by the increased CD133 expression. Notably, these results are in agreement with the evidence derived from RNAi-mediated downregulation of mTOR expression. These data suggest that mTOR signaling is involved in regulation of the balance of proliferation and differentiation of CSCs in Ras-dependent tumorigenesis and that transient inhibition of mTOR promotes tumor re-emergence via an increased CD133+ subpopulation.

Taken together, our observations show that mTOR inhibition enriches CD133+ subpopulations. This enrichment is most likely achieved through blocking differentiation of the CD133+ subpopulations, enhancing apoptosis in the CD133- subsets, and triggering the conversion of CD133- to CD133+ cells. The maintenance of CD133+ cells *in vivo* by rapamycin leads to high continuous tumorigenic potential. Therefore, therapeutic design strategies should consider the possibility that complex regulatory events in CSC generation may be triggered by mTOR inhibition.

## Materials and Methods

### Ethics statement

All animal experiments were conducted in accordance with protocols approved by the Shanghai Medical Experimental Animal Care Commission (approval ID. ShCI-11-020).

### Cell culture

Isolation, culturing and retroviral infection of purified p53-/- mouse fetal liver progenitor cells were performed as described by Zender et al. [61], [62]. Retroviruses were based on MSCV-IRES-GFP vectors containing cDNAs encoding H-Ras and used for infection of the purified cells. H-Ras-transformed mouse liver tumor cells were cultured in a maintenance medium containing 1∶1 DMEM:F12 (GIBCO, Grand Island, NY) with 10% fetal bovine serum (GIBCO) and the following additives: dexamethasone (1×10^−7^ M; Sigma-Aldrich, St. Louis, MO), nicotinic acid (0.01 M; Sigma-Aldrich), 2-mercaptoethanol (50 mM; Sigma-Aldrich), 1× ITS liquid media supplement (Sigma-Aldrich), EGF (20 ng/ml; R&D Systems, Minneapolis, MN), HGF (50 ng/ml; R&D Systems), and penicillin/streptomycin (1% [v/v]; GIBCO). For treatment studies, cells were plated and grown overnight in 1∶1 DMEM:F12 with 10% fetal bovine serum and penicillin/streptomycin (1% [vol/vol]). The medium was then replaced the following morning with medium containing rapamycin (Cell Signaling Technology, Beverly, MA) at concentrations of 0 to 100 nM. To assess the inhibition of mTOR by rapamycin, cells were treated with rapamycin (10 nM) for 24 h. These cells were lysed for immunoblot analysis.

Human liver tumor cell lines, Huh7 (purchased from Riken Cell Bank, Tsukuba Science City, Japan), MHCC-97L (purchased from Liver Cancer Institute, Fudan University, Shanghai), Hep3B and PLC/PRF/5 (purchased from American Type Culture Collection, Manassas, VA) were maintained in DMEM with high glucose (GIBCO) supplemented with 10% fetal bovine serum and penicillin/streptomycin (1% [vol/vol]).

### Retroviral transfection

A Rheb cDNA was Flag-tagged and cloned into the retroviral expression vector MSCV (MSCV-Rheb). Recombinant retroviral particles were generated from the Phoenix packaging cell line and harvested 2 consecutive times for infection. Transfected cells were pooled for further analysis after selection with 10 µg/ml of puromycin (Sigma-Aldrich) for 1 week and then with 5 µg/ml for additional 1–2 weeks. Short-hairpin RNAs targeting *mTOR* (mTOR-sh1 and mTOR-sh2), based on *mTOR* coding sequences 5'-AAGAATGTTGACCAATGCT-3' and 5'-TGATGGACACAAATACCAA-3' respectively, were expressed from the LTR promoter of MSCV retroviruses.

### Immunoblotting

Cell lysates were prepared with RIPA lysis buffer (Beyotime, Nantong, China) with Protease Inhibitor Cocktail and PhosSTOP Phosphatase Inhibitor Cocktail (Roche, Monza, IT) added. Specific antibodies were used for analysis: anti-phospho-mTOR (Ser2448), anti-mTOR, anti-phospho-p70 S6 kinase (Thr389), anti-p70 S6 kinase (all from Cell Signaling Technology); anti-Flag (Sigma); and anti-α-tubulin (Santa Cruz Biotechnology, Santa Cruz, CA). Images were obtained with the ChemiDoc^TM^ XRS system (Bio-Rad Laboratories, Hercules, CA).

### RNA extraction, reverse-transcription, and RT-qPCR

Total RNA was extracted from cultured cells with RNAiso Reagent (TaKaRa, Dalian, China) and converted to cDNA using the PrimeScript^TM^ RT reagent kit (TaKaRa) according to the manufacturer’s protocol. Gene expression analysis was determined using primers for *CD133*, *AFP*, *albumin* (*Alb*), *cytokeratin 19* (*CK19*), and *GAPDH*, as listed in Table S1. For detection of *CD133* expression, relative quantitative PCR (qPCR) was performed on a 7300 Real-Time PCR System (Applied Biosystems, Foster City, CA) using Premix Ex TaqTM (TaKaRa). Quantitative real-time PCR was performed using SYBR green mix from TaKaRa for *Bmi1*, *Klf4*, *Nanog*, *Oct4*, and *Sox2*. All samples were normalized to the level of *GAPDH*. The primers are listed in Table S1.

### Fluorescence-activated cell sorting and single-cell analysis

Cells were dissociated and resuspended in PBS containing 0.5% BSA. Flow cytometry was performed with PE-conjugated anti-mouse CD133 antibody (eBioscience, San Diego, CA) or PE-conjugated anti-human CD133 antibody (Miltenyi Biotec, Bergisch Gladbach, Germany) incubated at 4°C for 30 min. Rat IgG1/κ antibody conjugated to phycoerythrin was used as an isotype control. Preincubation with 7-AAD (Sigma-Aldrich) was used to exclude dead cells.

For cell sorting, samples were stained with PE-conjugated anti-mouse CD133 antibody and analyzed using a MoFlo XDP (Beckman Coulter, Fullerton, CA) with rat IgG1/κ antibody conjugated to phycoerythrin as isotype controls. Isolated CD133- or CD133+ cells were plated in 6-well plates. For single-cell experiments, single CD133- or CD133+ cells were stringently gated, isolated using a FACSVantage set for Single Cell Purity, and robotically plated into a 96-well plate with 150 µl medium/well. After 24 h, an additional 50 µl medium with or without rapamycin was added to each well. After 1–2 weeks in culture, robust single-cell-derived colonies that filled more than 50% of the well area were transferred into 24-well plates for expansion.

### Tumor cell preparation

Tumor tissue was mechanically cut into small pieces with sterile scalpel blades, then enzymatically dissociated for 1 h with a mixture of collagenase type I (0.05 mg/ml; Sigma-Aldrich), collagenase type IV (0.05 mg/ml; Sigma-Aldrich), hyaluronidase (0.025 mg/ml; Sigma-Aldrich), and DNase I (0.01 mg/ml; Roche) at 37°C and shaken repeatedly. The dissociated sample was then filtered (70-µm pore size) and washed with PBS. Cells were then injected into nude BALB/c mice for secondary tumor propagation or stained with mouse CD133-PE antibody for assessing CD133 expression using a FACSCalibur flow cytometer (BD Biosciences, San Jose, CA).

### Subcutaneous implantation into nude BALB/c mice and rapamycin treatment

H-Ras-transformed mouse liver tumor cells (1×10^6^) were suspended in 100 µl PBS and injected subcutaneously into male nude BALB/c mice (5 to 6 weeks of age). Tumor size was serially measured using calipers. Tumor volume was estimated using the following formula: (*w*_1_×*w*_2_×*w*_2_)/2, where *w*_1_ represents the length and *w*_2_ represents the width of the tumor. In some experiments, rapamycin (LC Laboratories, Woburn, MA) was reconstituted in absolute ethanol at 10 mg/ml and diluted in 5% PEG-400 (Sigma-Aldrich) and 5% Tween-80 (Sigma-Aldrich) for treatment of mice. Each treatment group consisted of 4 or 5 animals. Nude mice bearing xenografts received 1.5 mg/kg rapamycin by i.p. injection daily starting 3 weeks after tumor transplantation. Control animals bearing tumors were treated with diluent at equivalent doses and schedules to the experimental animals. Tumors were removed and injected into secondary recipients by limiting dilution experiments.

### Cell viability assay

A total of 500 cells/well were plated in 96-well plates. After 24 h, rapamycin (10 nM) was added to the media. Cell viability was performed daily by adding 3-(4,5-dimethyl-2-thiazolyl)-2,5-diphenyl-2H-tetrazolium bromide (MTT) (Sigma-Aldrich) to each well and measuring the absorbance at 540 nm.

### Apoptosis assay

For quantification of apoptosis, Pharmingen™ PE Aneexin V Apoptosis Detection Kit (BD Biosciences) was performed according to the manufacturer’s instructions.

### Statistical analysis

Statistical analyses were performed using SPSS 11.5.0. A paired, two-tailed Student’s *t* test was used to determine the significance between two groups. A *p* value less than 0.05 was considered statistically significant.

## Supporting Information

Figure S1 **Progenitor cell phenotype and tumorigenesis in LPC-H and LPC-H12 cells.** **A** RT-PCR analysis revealed that both hepatocyte and cholangiocyte markers were expressed in LPC-H and LPC-H12 cells. **B** The tumorigenic potential of LPC-H and LPC-H12 cells. The tumor-forming ability of LPC-H12 cells was higher than that of LPC-H cells.
(TIF)

Figure S2 **Inhibition of mTOR up-regulates the proportion of CD133+ cells.** **A** Flow cytometer analysis of CD133 expression in LPC-H on days 3 and 6 after rapamycin treatment of different concentrations. **B** The bar graphs illustrate the sustained increase in CD133+ cells in LPC-H12. Data shown are the means±S.E.M. of the results from three experiments. **C** mTOR signaling was efficiently inhibited by rapamycin. Phosphorylated mTOR (p-mTOR), mTOR, p-p70 S6, and p70 S6 were measured by immunoblot, and α-tubulin was used as a loading control. **D** Sustained increases of CD133 expression were observed in LPC-H12 cells stably expressing shRNAs targeting mTOR. The means±S.E.M. of the percentages of CD133+ cells in LPC-H12 cells infected with retrovirus expressing mTOR-shRNA (n = 3) are shown.
(TIF)

Figure S3 **Selected sensitivity to rapamycin existed between CD133- and CD133+ cells.** **A** Rapamycin induced apoptosis in the CD133- and CD133+ compartments of LPC-H12 cells. **B** Cell proliferation in CD133- and CD133+ subsets in LPC-H12 cells after treatment with rapamycin (10 nM) for 5 days. Cell viability was measured by MTT.
(TIF)

Table S1 **List of the primers for RT-PCR.**
(DOC)
