# Supplementary figures and images for: Transient mTOR Inhibition Facilitates Continuous Growth of Liver Tumors by Modulating the Maintenance of CD133+ Cell Populations
Source: PLoS One. 2011 Dec 1;6(12):e28405. doi: 10.1371/journal.pone.0028405 (PMC3228748; doi:10.1371/journal.pone.0028405)

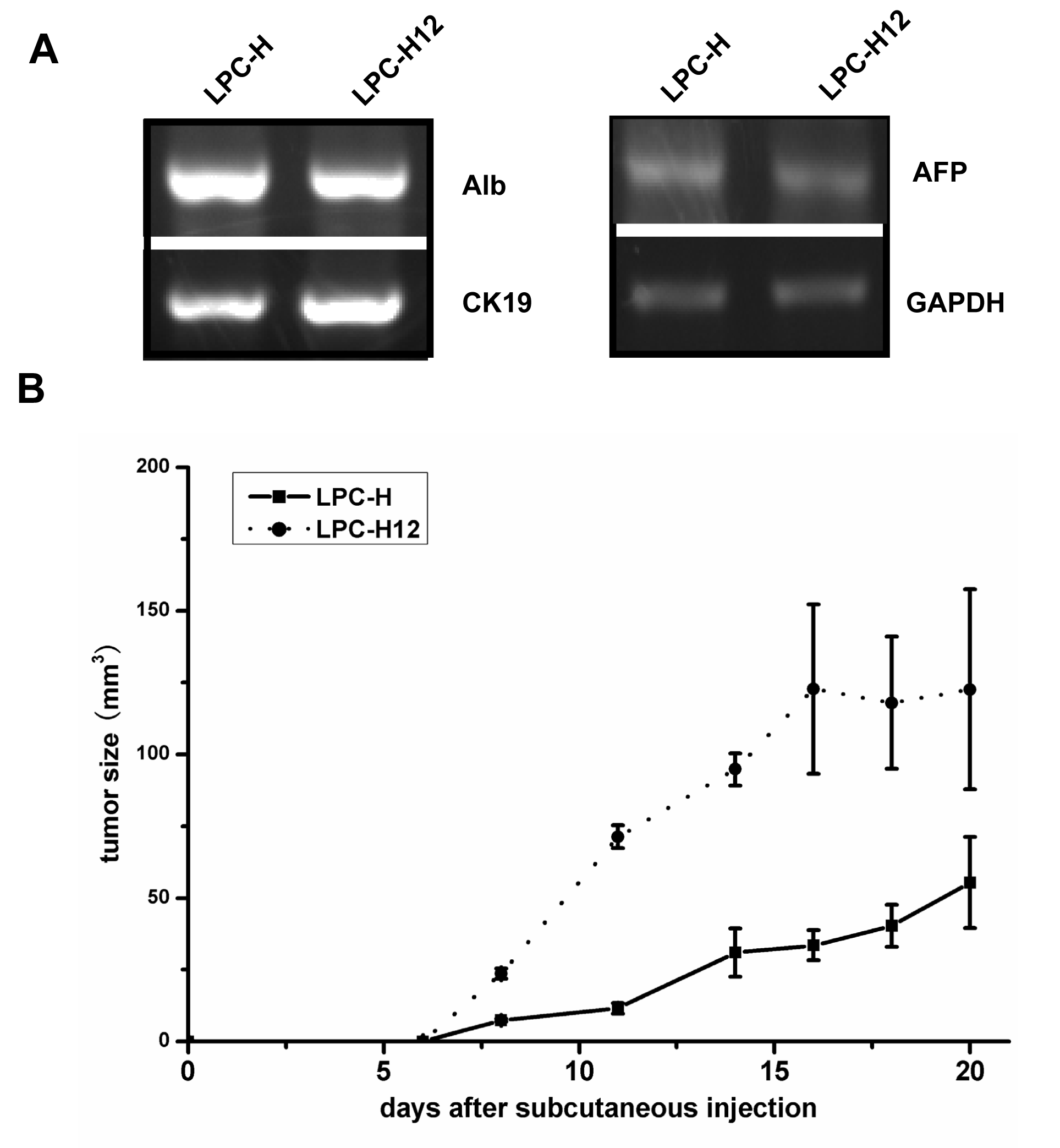

Supplement: Figure S1 — Progenitor cell phenotype and tumorigenesis in LPC-H and LPC-H12 cells. A RT-PCR analysis revealed that both hepatocyte and cholangiocyte markers were expressed in LPC-H and LPC-H12 cells. B The tumorigenic potential of LPC-H and LPC-H12 cells. The tumor-forming ability of LPC-H12 cells was higher than that of LPC-H cells. (TIF) [file pone.0028405.s001.tif]

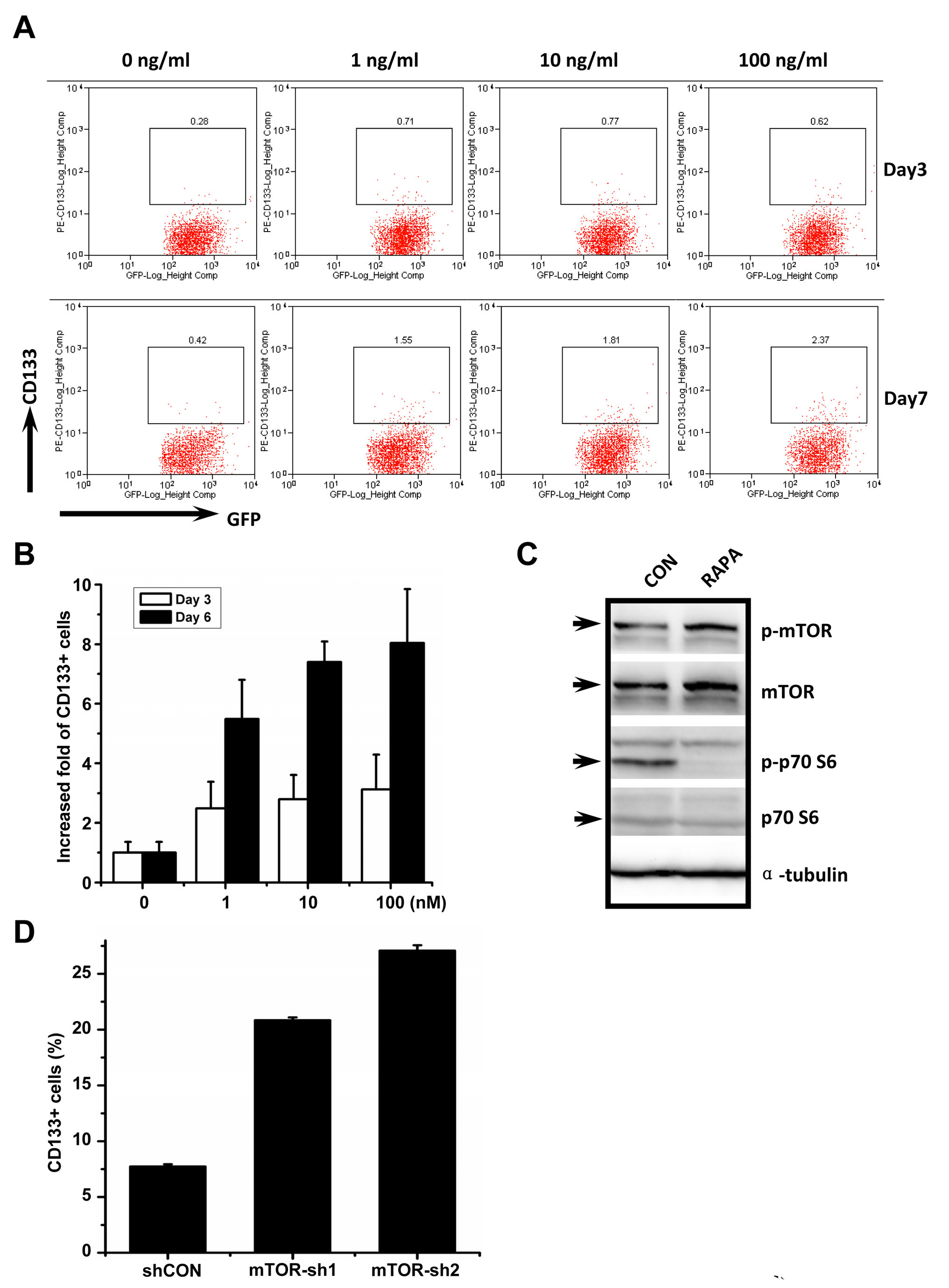

Supplement: Figure S2 — Inhibition of mTOR up-regulates the proportion of CD133+ cells. A Flow cytometer analysis of CD133 expression in LPC-H on days 3 and 6 after rapamycin treatment of different concentrations. B The bar graphs illustrate the sustained increase in CD133+ cells in LPC-H12. Data shown are the means±S.E.M. of the results from three experiments. C mTOR signaling was efficiently inhibited by rapamycin. Phosphorylated mTOR (p-mTOR), mTOR, p-p70 S6, and p70 S6 were measured by immunoblot, and α-tubulin was used as a loading control. D Sustained increases of CD133 expression were observed in LPC-H12 cells stably expressing shRNAs targeting mTOR. The means±S.E.M. of the percentages of CD133+ cells in LPC-H12 cells infected with retrovirus expressing mTOR-shRNA (n = 3) are shown. (TIF) [file pone.0028405.s002.tif]

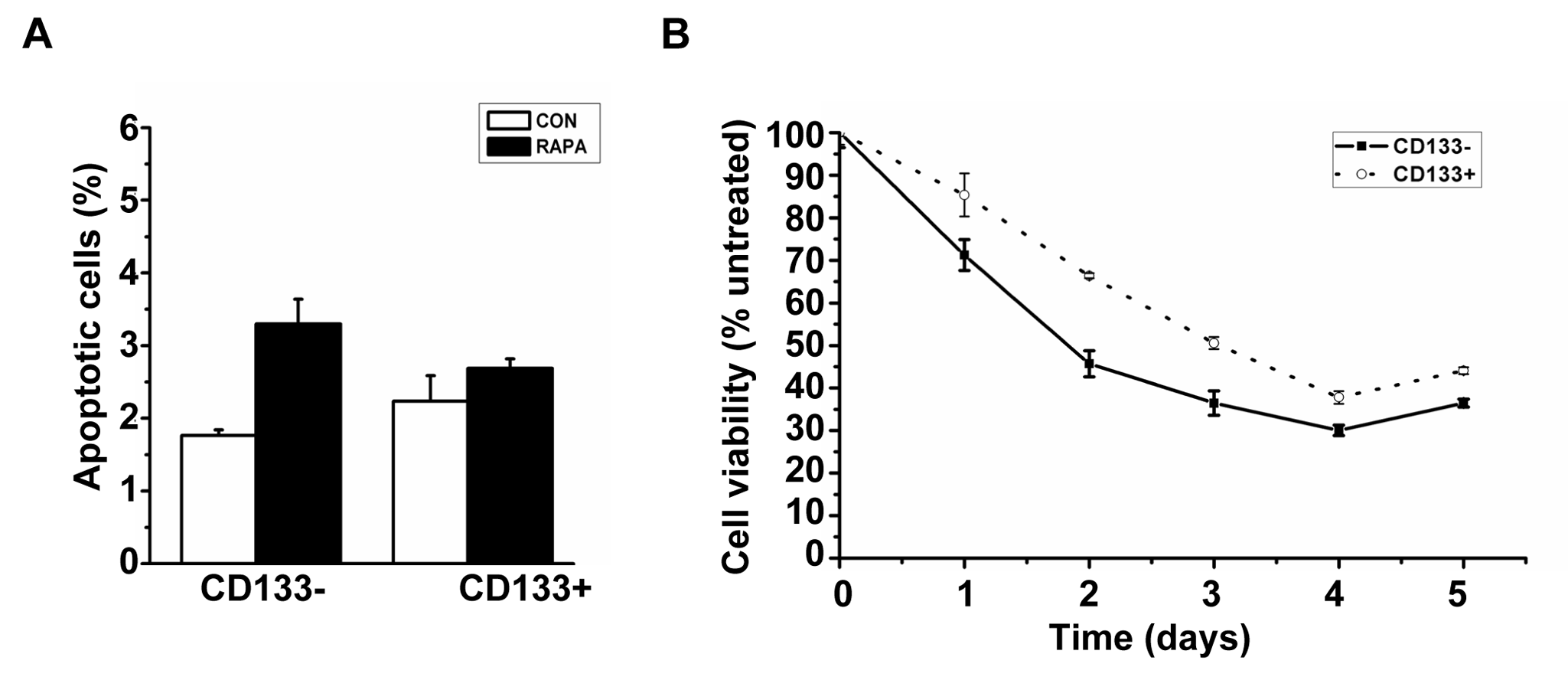

Supplement: Figure S3 — Selected sensitivity to rapamycin existed between CD133- and CD133+ cells. A Rapamycin induced apoptosis in the CD133- and CD133+ compartments of LPC-H12 cells. B Cell proliferation in CD133- and CD133+ subsets in LPC-H12 cells after treatment with rapamycin (10 nM) for 5 days. Cell viability was measured by MTT. (TIF) [file pone.0028405.s003.tif]
