# Supplementary material for: Transient mTOR Inhibition Facilitates Continuous Growth of Liver Tumors by Modulating the Maintenance of CD133+ Cell Populations
Source: PLoS One. 2011 Dec 1;6(12):e28405. doi: 10.1371/journal.pone.0028405 (PMC3228748; doi:10.1371/journal.pone.0028405)
Supplement: Table S1 — List of the primers for RT-PCR. (DOC) [file pone.0028405.s004.doc]

**Table S1. List of the primers for RT-PCR**

| **NAME** | **Primer (5'-3')** |  |
| --- | --- | --- |
| alb-1 | CATGACACCATGCCTGCTGAT | PCR for mouse albumin |
| alb-2 | CTCTGATCTTCAGGAAGTGTAC |
| afp-1 | ACTCACCCCAACCTTCCTGTC | PCR for mouse AFP |
| afp-2 | CAGCAGTGGCTGATACCAGAG |
| ck19-1 | GTCCTACAGATTGACAATGC | PCR for mouse CK19 |
| ck19-2 | CACGCTCTGGATCTGTGACAG |
| mCD133-1 | CTCTCCCTCCTGGTGATTTG | PCR for mouse CD133 |
| mCD133-2 | CAGTTTCTGGGTCCCTTTGA |
| mGAPDH-1 | GTCCCGTAGACAAAATGGTGA | PCR for mouse GAPDH |
| mGAPDH-2 | TGCATTGCTGACAATCTTGAG |
| mBmi-sybr-s | AAATTAGTCCCAGGGCTTTTCA | SYBR PCR for mouse Bmi1 |
| mBmi-sybr-a | GCAACTTCTCCTCGGTCTTCAT |
| mMyc-sybr-s | TGACCTAACTCGAGGAGGAGCTGGAATC | SYBR PCR for mouse c-Myc |
| mMyc-sybr-a | AAGTTTGAGGCAGTTAAAATTATGGCTGAAGC |
| mNanog-sybr-s | GCACTCAAGGACAGGTTTCAGA | SYBR PCR for mouse Nanog |
| mNanog-sybr-a | GCACTTCATCCTTTGGTTTTGA |
| mKlf4-sybr-s | AAAAGAACAGCCACCCACACTT | SYBR PCR for mouse Klf4 |
| mKlf4-sybr-a | CCCAGTCACAGTGGTAAGGTTTC |
| mOct4-sybr-s | TCTTTCCACCAGGCCCCCGGCTC | SYBR PCR for mouse Oct4 |
| mOct4-sybr-a | TGCGGGCGGACATGGGGAGATCC |
| mSox2-sybr-s | TAGAGCTAGACTCCGGGCGATGA | SYBR PCR for mouse Sox2 |
| mSox2-sybr-a | TTGCCTTAAACAAGACCACGAAA |
| mGAPDH-sybr-s | AGGTCGGTGTGAACGGATTTG | SYBR PCR for mouse GAPDH |
| mGAPDH-sybr-a | TGTAGACCATGTAGTTGAGGTCA |
| mCD133-qF | ACACCAACACCAAGAACAAGG | Taqman PCR for mouse CD133 |
| mCD133-qR | GACAGGAGTTACTTTGGGTTTTAG |
| mCD133-Probe | TCTCAGACCTGGATGGCATCGGCTC |
| mGAPDH-qF | CAATGTGTCCGTCGTGGATCT | Taqman PCR for mouse GAPDH |
| mGAPDH-qR | GTCCTCAGTGTAGCCCAAGATG |
| mGAPDH-Probe | CGTGCCGCCTGGAGAAACCTGCC |
| hBmi1-qF | GTTTCCTCACATTTCCAGTACTATG | Taqman PCR for human Bmi1 |
| hBmi1-qR | TTCCTTAACAGTCTCAGGTATCAAC |
| hBmi1-Probe | CCAGCAACAGCCCCAGCGGTAAC |
| hNanog-qF | CCAGCTGTGTGTACTCAATGATAG | Taqman PCR for human Nanog |
| hNanog-qR | ATTGCTATTCTTCGGCCAGTTG |
| hNanog-Probe | ACCTCAGCCTCCAGCAGATGCAAGA |
| hOct4-qF | GTGGTCCGAGTGTGGTTCTGT | Taqman PCR for human Oct4 |
| hOct4-qR | GCATAGTCGCTGCTTGATCG |
| hOct4-Probe | ACCGGCGCCAGAAGGGCAA |
| **NAME** | **Primer (5'-3')** |  |
| hCD133-qF | ACCCAACATCATCCCTGTTCTTG | Taqman PCR for human CD133 |
| hCD133-qR | GCTGGTCAGACTGCTGCTAAG |
| hCD133-Probe | CATGGCAACAGCGATCAAGGAGACC |
| hGAPDH-qF | GGACCTGACCTGCCGTCTAG | Taqman PCR for human GAPDH |
| hGAPDH-qR | TAGCCCAGGATGCCCTTGAG |
| hGAPDH-Probe | CCTCCGACGCCTGCTTCACCACCT |
